# A pupal transcriptomic screen identifies Ral as a target of store-operated calcium entry in *Drosophila* neurons

**DOI:** 10.1038/srep42586

**Published:** 2017-02-14

**Authors:** Shlesha Richhariya, Siddharth Jayakumar, Katharine Abruzzi, Michael Rosbash, Gaiti Hasan

**Affiliations:** 1 National Centre for Biological Sciences, Tata Institute of Fundamental Research, Bangalore 560065, India; 2 Manipal University, Manipal 576104, India; 3 Howard Hughes Medical Institute, National Center for Behavioral Genomics, Department of Biology, Brandeis University, Waltham, MA 02454, USA

## Abstract

Transcriptional regulation by Store-operated Calcium Entry (SOCE) is well studied in non-excitable cells. However, the role of SOCE has been poorly documented in neuronal cells with more complicated calcium dynamics. Previous reports demonstrated a requirement for SOCE in neurons that regulate *Drosophila* flight bouts. We refine this requirement temporally to the early pupal stage and use RNA-sequencing to identify SOCE mediated gene expression changes in the developing *Drosophila* pupal nervous system. Down regulation of *dStim*, the endoplasmic reticular calcium sensor and a principal component of SOCE in the nervous system, altered the expression of 131 genes including *Ral*, a small GTPase. Disruption of Ral function in neurons impaired flight, whereas ectopic expression of *Ral* in SOCE-compromised neurons restored flight. Through live imaging of calcium transients from cultured pupal neurons, we confirmed that Ral does not participate in SOCE, but acts downstream of it. These results identify neuronal SOCE as a mechanism that regulates expression of specific genes during development of the pupal nervous system and emphasizes the relevance of SOCE-regulated gene expression to flight circuit maturation.

Calcium is a key secondary messenger in metazoan cells where it regulates a number of cellular processes^1^. Specifically in excitable cells, calcium regulates their excitability, neurotransmitter release^2^, neurotransmitter specification^3^ and dendritic development^4^ amongst other processes. The specificity of calcium signals arises from different spatial and temporal signatures of the different modes of calcium entry into a cell^5^. Store-operated calcium entry (SOCE) is a sustained rise in cytosolic calcium in response to depletion of the endoplasmic reticular stores^6^. SOCE through the endoplasmic reticular calcium sensor STIM^7^ and the Calcium Release Activated Calcium (CRAC) channel Orai^8^,^9^ is the major source of calcium entry in non-excitable lymphocytes and is essential for their activation and cytokine gene expression^10^. SOCE through the STIM/Orai pathway has also been observed in invertebrate^11^ and vertebrate^12^,^13^ neurons. Although a recent study has implicated it in synaptic transmission^14^, the cellular functions of SOCE in neurons are still not well understood.

In *Drosophila* neurons, both SOCE components, dSTIM and dOrai, are required for flight^11^. dOrai is required in dopaminergic neurons during flight circuit development in pupae^15^. SOCE requirement is thus temporally distinct from the observable flight phenotype, indicating that SOCE is required in neurons during maturation of the flight circuit in pupae^16^. That said, the cellular and molecular processes underlying development and maturation of the flight circuit in pupae, remain largely elusive.

Regulation of gene expression in the developing flight circuit is a possible mechanism of SOCE action in pupae. Transcriptional regulation by SOCE was recently suggested in vertebrate neural progenitor cells^12^. Moreover, we recently used a candidate gene approach in a study of dopaminergic neurons from pupae and identified SOCE-dependant changes in expression levels of mRNAs encoding the dopamine synthesizing enzyme Tyrosine Hydroxylase (*pale* or TH), the Dopamine Transporter (*DAT*) and a voltage-gated calcium channel subunit (*cacophony*)^15^. However, flight deficits obtained by knockdown of these genes in dopaminergic neurons do not fully explain the stronger flight deficits observed upon SOCE knockdown in all neurons^11^, suggesting a more general effect of SOCE on neuronal gene expression profiles.

In this study, we identify a 24 h window during pupal development when dSTIM is most critical for flight. High-throughput transcriptomic analyses within the identified 24 h window and 36 h later indicated a greater contribution of age rather than SOCE, to the observed expression differences. In the early transcriptomic screen, concurrent with the requirement of dSTIM for flight, we identified 131 genes whose expression levels change upon knockdown of *dStim*. Amongst these genes, the function of *Ral*, which encodes a small GTPase, was investigated further by molecular, cellular and behavioural genetic studies.

## Results

### dSTIM requirement for flight is restricted to the early pupal stages of *Drosophila* development

**Figure 1:**
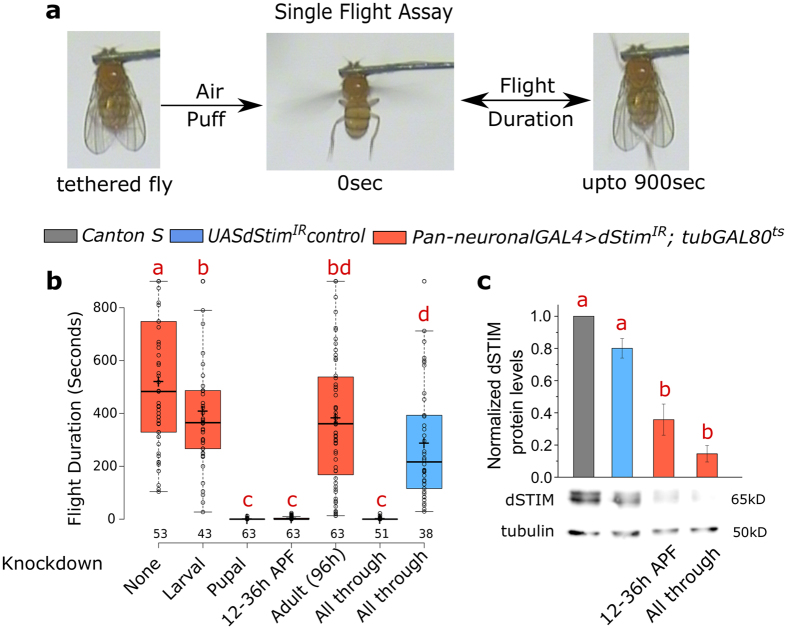
dSTIM is required in the nervous system during early pupal development for flight. (**a**) Schematic of the assay used for measuring duration of flight bouts. (**b**) Duration of flight bouts in flies with pan-neuronal knockdown of *dStim* at different developmental stages is plotted as box plots. Stages of *dStim* knockdown are indicated below each box. Stage specific knockdowns were performed with the TARGET system, where a *GAL80*^*ts*^ transgene was driven by the *tubulin* promoter (*tubGAL80*^*ts*^). Horizontal lines in the box represent medians, crosses indicate the means, box limits indicate the 25th and 75th percentiles, whiskers extend 1.5 times the interquartile range from the 25th and 75th percentiles, individual data points are represented as open circles and the numbers below represent the number of flies tested for each box. (**c**) Bars represent dSTIM protein levels (means ± SEM) normalized to Tubulin in protein lysates isolated from CNS of the indicated genotypes. A representative western blot used for quantification is shown below. n ≥ 3. Red alphabets over the box plots/bar graphs represent statistically indistinguishable groups (one-way ANOVA with a post hoc Tukey’s test p < 0.05). APF- After Puparium Formation.

SOCE in the central nervous system (CNS) is essential for *Drosophila* flight^11^. Single genes encode the ER calcium sensor dSTIM and the SOCE channel, dOrai in the *Drosophila* genome^8^,^17^. To determine the temporal requirement for SOCE in flight, we down regulated *dStim* gene expression with an RNAi transgene and the pan-neuronal GAL4 driver *elav*^*C155*^ at different stages of development by raising the rearing temperature to 29 °C using the TARGET system^18^. Knockdown of *dStim* was chosen over *dOrai*, primarily because the *dStim* RNAi (*dStim*^*IR*^) strain is both specific and more effective compared to the *dOrai* RNAi strains^11^,^19^. Adult flies were subsequently tested for their ability to maintain flight over several minutes by the single flight assay^15^ (Fig. 1a). Knockdown of *dStim* exclusively in pupal stages rendered the flies almost flightless. The time-window for requirement of dSTIM was further narrowed to 12 h–36 h after puparium formation (APF), when knockdown of *dStim* resulted in near complete loss of flight, similar to what was observed upon either pupal knockdown or knockdown throughout development (Fig. 1b). Control flies without the *GAL4* but only the *UAS-dStim*^*IR*^ transgene when subjected to the elevated temperature throughout, exhibited near normal flight (Fig. 1b), confirming that the flight phenotype is due to neuronal knockdown of *dStim*. Both *dStim* RNA and dSTIM protein levels were significantly reduced by the short 24 h knockdown between 12 h–36 h APF (Figs 1c and S1).

### *dStim* knockdown in pupae results in altered gene expression

**Figure 2:**
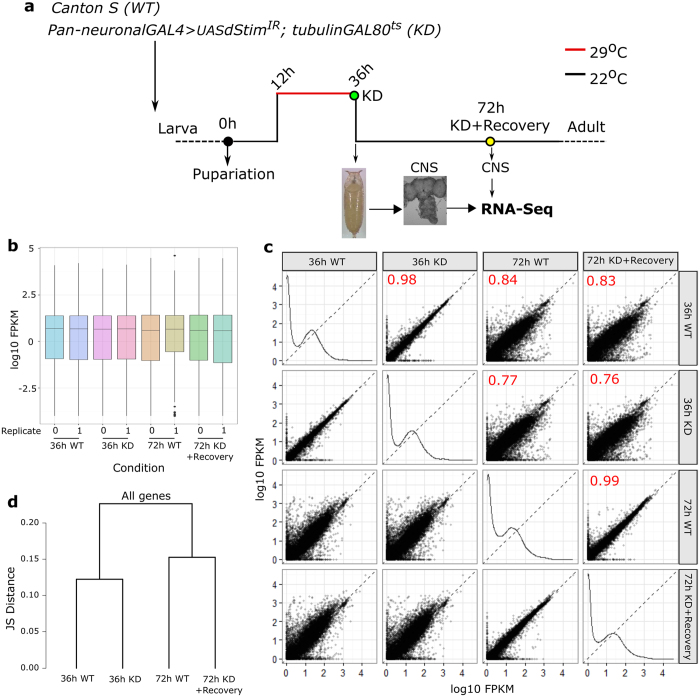
RNA-seq at two pupal time points reveals a larger contribution of age over *dStim* knockdown to gene expression. (**a**) Schematic representation of the experimental design for RNAseq. Pan- neuronal *dStim* knockdown was for 24 h from 12 h–36 h APF, following which the transcriptome was sequenced from wild type and knockdown CNS. Another RNAseq was performed at 72 h APF, allowing 36 h of recovery post *dStim* knockdown. (**b**) Box plots indicating the distribution of reads across all the samples sequenced. (**c**) Scatter plots of all the four conditions sequenced against each other. Each dot on these plots represents a single gene. The numbers in red indicate correlation coefficient (R^2^) values between the two conditions. Samples at the same age correlate better than across age. (**d**) A dendrogram of Jensen-Shannon divergences analysing pattern of gene expression between the indicated conditions.

The pupal specific requirement of dSTIM for adult flight supports a role for SOCE in flight circuit development and agrees with previous observations of a pupal requirement for *dOrai* during maturation of the *Drosophila* flight circuit^15^. To determine if *dStim* knockdown and reduced SOCE alter neuronal gene expression profiles, we performed high throughput transcriptomic screens from wild type and *dStim* knockdown pupal CNS at two stages of pupal development. The first was at 36 h APF post *dStim* knockdown for a period of 24 h (12 h–36 h APF), when *dStim* is required most critically for flight (Figs 1b and 2a). The restricted period of knockdown enabled the capture of primary changes in gene expression and helped minimize changes in gene expression due to secondary or tertiary effects. The second stage chosen was at 72 h APF following a recovery period of 36 h post knockdown to distinguish between transient and persistent changes (Fig. 2a). Biological duplicates were performed for each sample and an average of 14–20 million reads per sample were obtained, of which 87–90% mapped to the *Drosophila* genome. The distribution of reads looked uniform in all samples (Fig. 2b). As expected, the expression profile of wild type pupal CNS changed significantly from 36 h to 72 h. This difference was much greater than the effect of *dStim* knockdown on either of the stages (Fig. 2c) confirming that *dStim* knockdown does not alter global transcription levels. The expression profiles of wild type and *dStim* knockdown clustered together at the two time points (Fig. 2d).

Two independent methods, CuffDiff^20^ and EdgeR^21^ were used for differential expression analysis. These methods differ in their mode of normalization and the statistical tests used for differential expression. Hence they differ in their estimation of differentially expressed genes^22^,^23^. To introduce stringency, in our subsequent studies we considered genes that were identified as differentially expressed by both methods (for details, see Methods).

**Figure 3:**
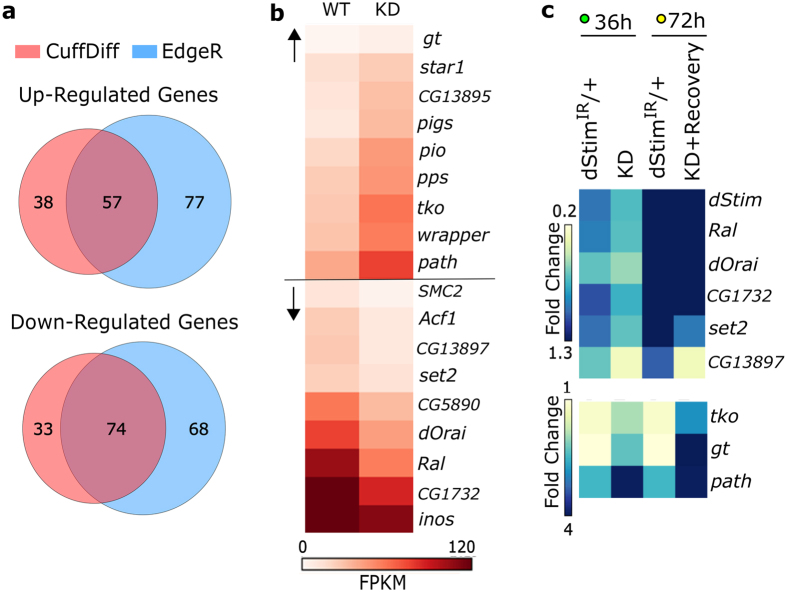
Altered gene expression upon *dStim* knockdown in early pupae. (**a**) Venn Diagrams representing the number of up and down regulated genes at 36 h APF as quantified by CuffDiff and EdgeR. (**b**) Heat map representing normalized read counts of some of the differentially expressed genes in wild type and knockdown conditions. FPKM - Fragments Per Kilobase per Million reads. (**c**) Heat map representing fold changes of the indicated genes, as measured by qPCR at the two time points of 36 h and 72 h, normalized to wild type levels (fold change = 1; not shown).

At the 36 h time point, 202 and 276 genes were identified by CuffDiff and EdgeR respectively, as differentially regulated between wild type and *dStim* knockdown. 131 genes were differentially regulated by both criteria, amongst which 57 were up and 74 were down regulated (Fig. 3a, Supplementary File 1). Specific GO categories do not appear to be enriched amongst the differentially regulated genes at this stage (Fig. S2a). Differential expression of selected genes across GO categories is shown in (Fig. 3b). They include the transcription factor *giant*, an amino acid transporter *pathetic*, a mitochondrial ribosomal protein *technical knockout* (up-regulated), the SOCE/calcium-release activated calcium (CRAC) channel *dOrai*, the H3K36 methyltransferase *dSet2*, a GABA transporter *CG1732 (Gat*) and a small GTPase *Ral* (down-regulated). Differences in expression for some of the up and down-regulated genes were further confirmed by quantitative PCR (Fig. 3c).

At 72 h APF, post knockdown and recovery, differentially expressed genes identified by CuffDiff and EdgeR varied significantly, with 54 and 367 genes respectively. Among these only 8 up and 9 down regulated genes were common (Fig. S2c) and the expression levels of 14 such genes did not recover from 36 h to 72 h despite the recovery of *dStim* expression (Fig. S2d,e), suggesting their regulation by additional mechanisms. Genes with apparently longer-term changes in expression include *dSet2* and *tko* (Fig. S2e and Fig. 3c). In contrast and like *dStim*, the expression of *dOrai, CG1732* and *Ral* was restored after recovery at 72 h, also confirmed by quantitative PCR (Fig. 3c).

### *Drosophila* flight requires *Ral* expression in neurons

Diverse classes of genes were affected by *dStim* knockdown and many of them could be potential regulators of flight. Because we identified a requirement for SOCE in flight during pupal development we focussed on genes with previously defined functions in developing neurons. One of the down-regulated genes, *Ral (dRal, Rala, CG2849*) is a small GTPase of the Ras superfamily and is the single *Drosophila* ortholog for the mammalian *RalA* and *RalB* genes^24^. Mammalian and *Drosophila* Ral are known to affect multiple functions in neural cells like exocytosis^25^,^26^,^27^, neurite branching^28^,^29^, and neuronal polarity^30^. Hence, we addressed Ral function in the context of *Drosophila* flight.

**Figure 4:**
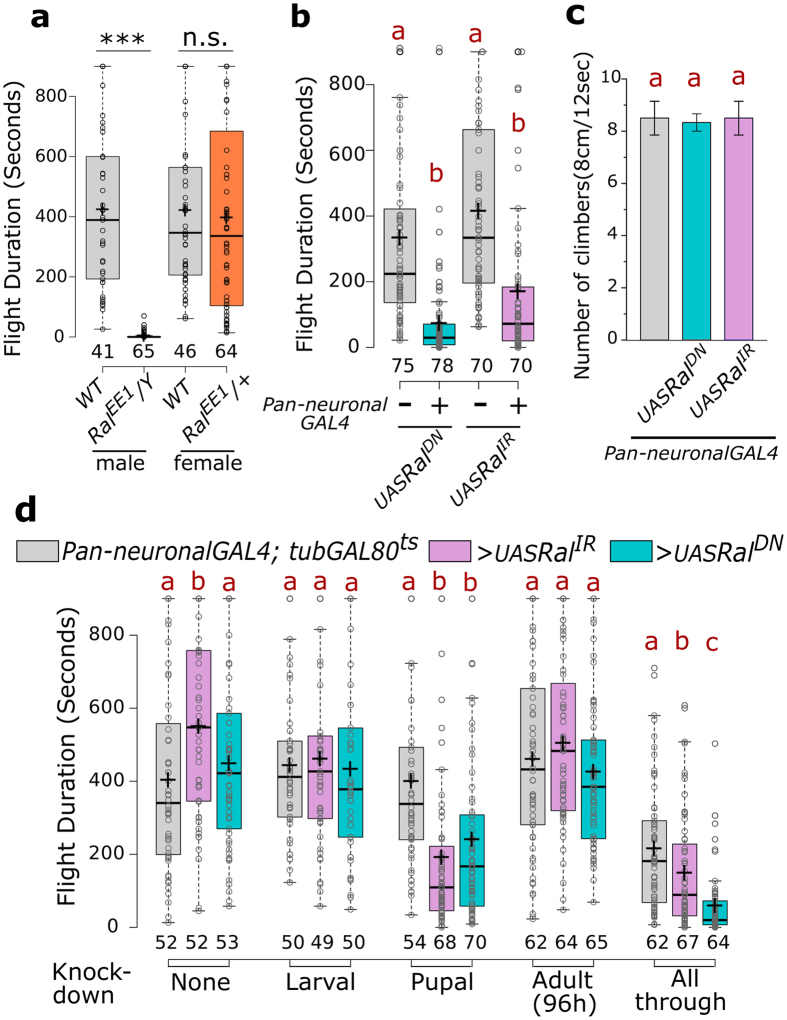
*Drosophila* flight requires *Ral* expression in neurons. (**a**) Box plots of flight bout durations of wild type and *Ral*^*EE1*^ flies. The males were hemizygous and females heterozygous for the mutation. ***p < 0.0001, n.s. not significant p = 0.6617, two-tailed t-test. (**b**) Box plots of flight bout durations of flies upon pan-neuronal expression of the dominant negative form of Ral (*Ral*^*DN*^) and an RNAi against Ral (*Ral*^*IR*^), along with corresponding UAS-controls. (**c**) Bars represent the climbing ability of flies, measured as the average number of flies (out of 10) that climb 8 cm in 12 seconds, upon pan-neuronal expression of *Ral*^*DN*^ and *Ral*^*IR*^. Error bars represent SEM calculated from three or more experiments. (**d**) Box plots of flight bout durations of flies upon pan-neuronal expression of *Ral*^*DN*^ *and Ral*^*IR*^ at the indicated stages of development using the TARGET system. Box plots and their symbols are as described for Fig. 1b. Red alphabets over the box plots/bar graphs represent statistically indistinguishable groups (one-way ANOVA with a post hoc Tukey’s test p < 0.05).

Viable males of the Ral mutant, *Ral*^*EE1*^, which harbours a single missense mutation S154L, are sterile^31^. When tested for flight they were identified as flight defective, whereas heterozygous females exhibit normal flight (Fig. 4a). The S25N mutation in *Ral* functions as a dominant negative and reduces Ral function^24^,^32^,^33^. Flies expressing *UAS-Ral*^*S25N*^ (henceforth referred to as *Ral*^*DN*^) with a pan-neuronal driver exhibit normal wings (Fig. S3) but significantly reduced flight times as compared to the *UAS-Ral*^*DN*^ control flies (Fig. 4b). Similarly, knockdown of *Ral* in the nervous system by means of an RNAi (*Ral*^*IR*^) also significantly shortened the duration of flight when compared to *UAS-Ral*^*IR*^ flies (Fig. 4b). The climbing ability of flies with compromised *Ral* levels or function in neurons appeared normal and agrees with previous data where reduced SOCE was found to affect flight but no other motor functions^15^ (Fig. 4c). Akin to manipulations of dSTIM levels, expression of either *Ral*^*DN*^ or *Ral*^*IR*^ exclusively in the pupal nervous system led to flight defects. Similar manipulations solely in the larval stage or for 4 days in the adult stage did not reduce the duration of flight bouts (Fig. 4d). These data confirm a requirement for Ral in pupal development of the *Drosophila* flight circuit.

### Ral acts downstream of intracellular calcium to regulate flight

**Figure 5:**
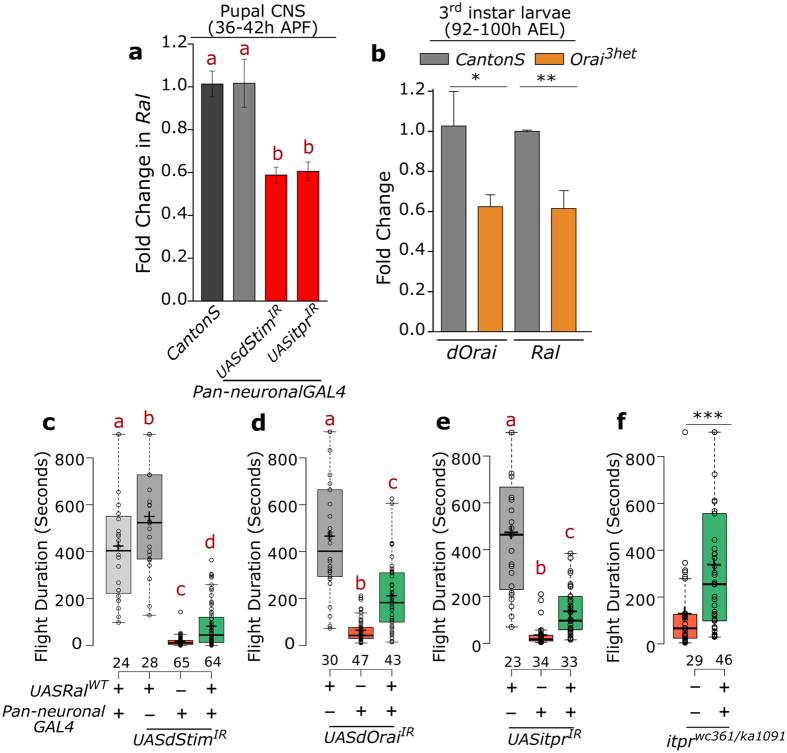
Over-expression of Ral can rescue flight deficits caused by loss of intracellular calcium signalling. (**a**,**b**) Fold change of *Ral* RNA levels obtained by qPCR from the indicated tissues, genotypes and ages represented as bars with error bars indicating SEM. (**c**,**d**) Box plots show the flight bout durations of female flies of the indicated genotypes. Over-expression of *Ral*^*WT*^ can partially rescue flight upon knockdown of the SOCE components *dStim* and *dOrai*. (**e**) Reduced flight bout durations in IP_3_R knockdown (*itpr*^*IR*^) and (**f**) mutant (*itpr*^*wc361*/*ka1091*^) strains can be rescued by over-expression of *Ral*^*WT*^. Box plots and their symbols are as described for Fig. 1b. Red alphabets over the box plots represent statistically indistinguishable groups (one-way ANOVA with a post hoc Tukey’s test p < 0.05). *p = 0.096, **p = 0.0073, ***p = 0.001; two-tailed t-test.

The transcriptomic screen identified *dStim* as a positive regulator of *Ral* expression. We further tested if a similar effect on Ral levels was obtained upon knockdown of the inositol trisphosphate receptor (IP_3_R), the calcium channel on the ER membrane encoded by *itpr* that also regulates SOCE in *Drosophila* neurons^11^. A significant reduction in Ral levels was obtained upon knockdown of *itpr* in pupal neurons (Fig. 5a), indicating a general effect downstream of the SOCE pathway. Larval levels of Ral were also reduced in heterozygous *dOrai*^3^ mutant animals (Fig. 5b), suggesting that the regulation of Ral expression by SOCE was not restricted to neurons or to the pupal stage.

To determine if reduced flight durations obtained by abrogating Ral function occur downstream of SOCE, we generated a fly strain with the coding sequence of *Ral* under UAS control (*Ral*^*WT*^). Expression of *Ral*^*WT*^ in the nervous system of either *dStim* or *dOrai* knockdown animals significantly rescued flight durations of female flies (Fig. 5c,d). As flies with overexpression of *Ral*^*WT*^ in the wild type background have little or no effect on flight duration (Figs 5c and S4a), deficits in flight circuit maturation by reduced SOCE in pupae can be overcome to a significant extent by *Ral* over-expression.

*itpr* also regulates flight circuit development^34^. Flight defects caused by *itpr* knockdown in the nervous system of female flies were also rescued by expression of *Ral*^*WT*^ (Fig. 5e).

Stronger flight defects in males, very likely because of insertion of the *elav*^*C155*^*GAL4* transgene on the X chromosome and concomitant dosage compensation^35^, were only marginally rescued by over-expression of *Ral*^*WT*^ (Fig. S4a,b,c,e). However, flight defects of a hetero-allelic *itpr* mutant *itpr*^*ka1091*/*wc361*^^34^ were significantly rescued in both sexes (Figs 5f and S4d), ruling out a sex-specific role for *Ral* in flight.

### Ral does not mediate SOCE

**Figure 6:**
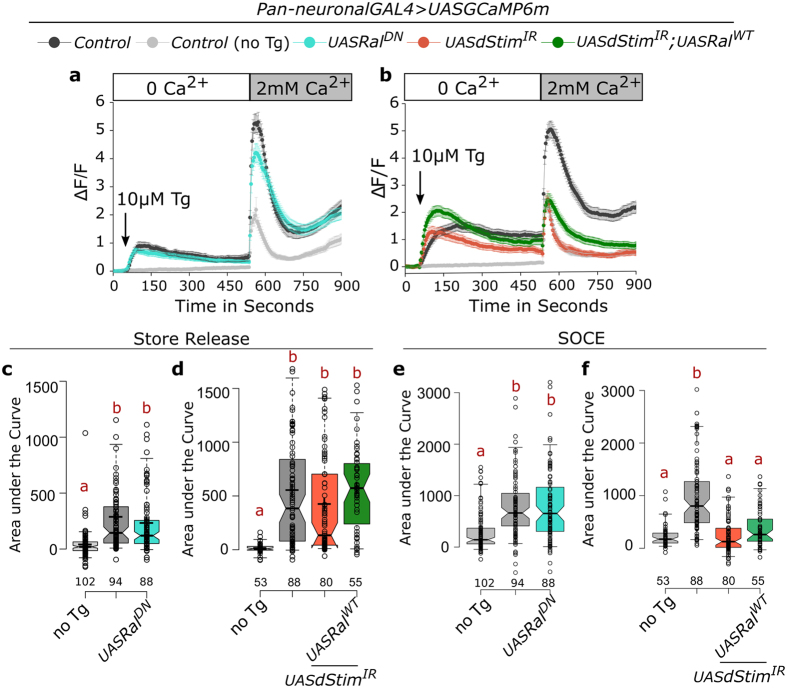
Ral does not mediate SOCE. (**a**,**b**) Traces represent mean (±SEM) of normalized changes in fluorescence (ΔF/F) of the Ca^2+^ sensor GCaMP6m over the indicated time periods. Fluorescence measurements were from cultured pupal neurons of the indicated genotypes. Treatment with thapsigargin (Tg) leads to passive release of ER store Ca^2+^ and extracellular addition of Ca^2+^ results in Store-operated Ca^2+^ entry or SOCE. (**c**,**d**) ER-store Ca^2+^ release, as estimated by area under the curve from 60 to 540 seconds represented as box plots. (**e**,**f**) SOCE, estimated by the area under the curve from 541 to 900 seconds is represented as box plots for the indicated genotypes. In the box plots, centre lines show the medians, crosses indicate the means, box limits indicate the 25th and 75th percentiles, whiskers extend to the 5^th^ and 95^th^ percentiles, individual data points are represented as open circles and the numbers below represent the n for each box. Notches represent 95% confidence interval for each median. Red alphabets over the box plots represent statistically indistinguishable groups (one-way ANOVA with a post hoc Tukey’s test p < 0.05).

Though unlikely, a possible mechanism by which Ral function could affect flight, is through SOCE itself. To test this, we cultured pupal neurons expressing the genetically encoded calcium sensor GCaMP6m^36^. Treatment with thapsigargin, an inhibitor of the sarco-endoplasmic Ca^2+^ -ATPase pump, in Ca^2+^ free media lead to an increase in cytosolic calcium levels, due to passive release from the ER stores in control neurons (Fig. 6a,c). Addition of calcium to the media led to a sustained rise in cytosolic calcium due to SOCE (Fig. 6a,e). Expression of *Ral*^*DN*^ in the neurons did not alter either passive ER-Ca^2+^ store release or SOCE (Fig. 6a,c,e). Passive ER-Ca^2+^ release in pupal neurons with *dStim* knockdown was not different from controls (Fig. 6b,d). The reduced SOCE observed upon *dStim* knockdown remained low both in presence and absence of *Ral*^*WT*^ over-expression (Fig. 6b,f). Expression of *Ral*^*WT*^ thus failed to rescue SOCE in *dStim* knockdown neurons, further supporting Ral function downstream of SOCE in pupal neurons.

## Discussion

A high-throughput transcriptomic screen was performed in pupal neurons to identify genes whose expression is dependent on SOCE. Among transcripts that were acutely down regulated, one encodes the small GTPase Ral. Similar to flight deficits observed by knockdown of the SOCE molecule dSTIM in pupal neurons, *Ral* mutants exhibit shorter flight bouts. We propose that neuromodulatory signals stimulate SOCE and consequently regulate the expression of genes required for multiple aspects of flight circuit maturation during pupal development. Although other SOCE-regulated genes very likely contribute to the observed SOCE-mediated flight deficits, our genetic experiments indicate that contribution of Ral to flight circuit development is substantial (Fig. 3a,b).

### Regulation of gene expression by SOCE in neurons

Regulation of gene expression by SOCE is well established in non-excitable cells^10^. Besides a few recent reports^12^,^15^, a similar role for SOCE in neurons is uncertain. Here we demonstrate that upon acute knockdown of the key SOCE molecule dSTIM in the pupal nervous system of *Drosophila*, specific gene expression changes occur. A primary consequence of *dStim* knockdown is reduced SOCE, though reduced basal cytosolic calcium levels in *Drosophila* neurons has also been reported^11^. Although changes due to secondary effects cannot be ruled out completely, gene expression changes that are observed from knockdown of *dStim* in the 24 h time window, and that are restored after 36 h, are very likely a primary result of reduced SOCE.

Most gene expression affected by *dStim* knockdown is restored after 36 h, but the expression level of a few genes remains altered over longer periods. Regulation of gene expression by SOCE thus appears to be multi-layered. Factors that regulate both acute and long-term effects of SOCE on gene expression in *Drosophila* neurons remain to be identified. The Nuclear factor of activated T-cells (NFAT) is activated by calcineurin post SOCE and is primarily responsible for changes in gene expression in mammalian cells^37^. Of the five NFAT proteins in mammals, NFAT1-4 is activated by calcineurin and thus changes in cellular calcium. In contrast NFAT5, the only form present in *Drosophila*, lacks the calcineurin binding site and is consequently unresponsive to calcium^38^. However, the fly homolog of another class of calcineurin-regulated transcription factors, transducers of regulated CREB activity (TORCs)^39^,^40^, also known as CREB-regulated transcription co-activator (Crtc), can be activated by intracellular calcium in *Drosophila* intestinal stem cells^41^. Moreover, Adf-1, belonging to the Myb family of transcription factors, acts downstream of the cellular calcium effector CaMKII to regulate dendritic growth^42^. Other transcription factors that regulate transcription in a calcium-dependant manner also exist^43^. A careful analysis to identify transcription factors downstream of SOCE in *Drosophila* neurons is therefore warranted.

### Ral as a regulator of flight circuit development

Our study defines a physiological role for Ral in pupal neurons that ultimately regulate flight duration in adults. However, its precise cellular role in this context is unclear. Ral has many known cellular functions, any or all of which could be relevant for flight circuit development. RalA and RalB regulate GTP-mediated exocytosis in mammalian neurons^25^,^26^. In *Drosophila* S2 cells too, delivery of secretory vesicles to the plasma membrane is reduced upon *Ral* knockdown^27^. Neurotransmitter release from synaptic vesicles during circuit development could affect synapse formation or strength as well as dendritic pruning^44^. A role for *Drosophila* Ral in exocytosis of neurotransmitters during synapse maturation and dendritic pruning in the developing pupal flight circuit^15^ is thus possible and needs further investigation. Ral is also known to affect receptor levels and thereby compromises post-synaptic function^45^,^46^,^47^. It is however, unlikely that SOCE and Ral regulate neurite branching in *Drosophila* pupae, as observed for RalA and B in cultured rat sympathetic neurons^29^. This is because compromising dOrai function in *Drosophila* dopaminergic neurons did not alter their neurite projections *in vivo*^15^.

### Other potential regulators of flight from the transcriptomic screen

Ral knockdown explains the flight defects of SOCE deficient flies to an extent, but over-expression of Ral rescued flight defects of *dStim* or *dOrai* knockdowns only partially (Fig. 5c). Therefore, other genes, with altered expression after *dStim* knockdown, are likely to contribute to the flight phenotype observed. Altered neurotransmitter levels at the synapse by changes in the expression of the GABA transporter (*CG1732*)^48^, which was also down-regulated upon *dStim* knockdown, could regulate flight bouts. Moreover, genes that regulate expression of other genes are good candidates for development and maturation of the flight circuit. One down-regulated gene, *dSet2* is a H3K36 methyltransferase and a positive regulator of gene transcription^49^. Another down-regulated gene, *Acf1* is a transcription factor implicated in dendritogenesis^50^. Interestingly, the transcript level of a known regulator of flight and the dSTIM partner, *dOrai* was also reduced upon *dStim* knockdown, indicating a feedback loop between *dStim* and *dOrai* expression.

Some genes like *dSet2* and *CG13987* remain down-regulated even after *dStim* expression is restored, suggesting that reduced SOCE leads to prolonged and indirect changes in gene expression of pupal neurons. At this point, it is unclear if such persistent changes are important for flight circuit development, subsequent to the requirement of SOCE, or if they are just changes that occur upon SOCE knockdown and cannot be reversed later.

Gene expression changes in pupae might also regulate flight by affecting wing development. In fact, a down-regulated gene *hinge3*/*CG13897* is a regulator of wing development^51^ and two other genes identified from the screen, *CG11382* and *CG11226* have been implicated in wing morphogenesis^52^. Wing posture is affected in a variety of *itpr* mutants^34^ as well as in flies with compromised neuronal SOCE^11^,^53^, thus making such SOCE-regulated genes interesting candidates for further investigation. It should be noted however that reduced Ral expression and function during pupal development did not alter gross wing morphology (Fig. S3).

Different genes might regulate specific aspects of flight circuit development, most likely in different cells. The exact identity of central neurons that regulate flight durations is not known. However reduced SOCE in dopaminergic^15^, peptidergic Dilp2 producing^53^ and glutamatergic neurons^11^ can either reduce flight times^11^,^53^ or abolish flight altogether^15^. Thus, the duration of flight bouts is affected not only by activity of flight motor neurons and neurons that connect to them directly as part of the central pattern generator, but also through neuromodulatory signals from central brain centres that can affect both flight circuit maturation and function. Further work is required to parse the regulation of gene expression by SOCE in different classes of such modulatory neurons.

### SOCE regulated gene expression: implications beyond flight

In light of SOCE-regulated Ral expression, a broader role for SOCE in neural circuit maturation and long-term function is likely. SOCE in neuronal cells has been implicated in a variety of developmental functions such as synaptic plasticity and axon guidance^54^. It is possible that SOCE-regulated gene expression changes, including altered Ral expression, mediate such functions. Moreover, de-regulation of calcium signalling in adults is associated with several neurodegenerative disorders like ataxia^55^, Parkinson’s^56^ and Alzheimer’s disease^57^,^58^. SOCE-regulated gene expression may thus contribute to other functions of SOCE in the adult nervous system, for example neuronal survival and neurodegeneration.

## Methods

### Fly rearing and stocks

*Drosophila* strains were grown on cornmeal medium supplemented with yeast. Flies from crosses involving *Ral*^*DN*^ and *Ral*^*IR*^ strains were reared at 29 °C in all cases. All other flies were reared at 25 °C unless stated otherwise. *Canton S* was used as wild-type (WT) throughout. All other fly stocks are listed in Supplementary Table 1.

### Single Flight Assay

Flies of either sex unless otherwise specified were aged for 3 to 5 days and were tested for flight by the single flight assay modified from^15^. Briefly, flies were anaesthetized on ice for a short time and then tethered between the head and thorax using a thin metal wire and nail polish. After allowing them to recover for ~15 minutes, a mouth blown air puff was given and flight time was recorded using a stop watch. Flight was recorded for a maximum of fifteen minutes for each fly in batches of 5–10 flies at a time. Flight times are represented as box plots generated using BoxPlotR^59^.

### RNA isolation and quantitative PCR

Central nervous systems (CNS) from pupae of the appropriate genotype and age were dissected in phosphate buffer saline prepared in double distilled water treated with diethyl pyrocarbonate (Sigma). CNS from 8–10 pupae were pooled per sample and homogenised in 500 μl TRIzol (Life Technologies) by vortexing immediately after dissection. After homogenization the sample was kept on ice and either processed within 30 min or stored at −80 °C for processing for up to 4 weeks. RNA was isolated by following manufacturer’s protocol for TRIzol. Purity of the isolated RNA was estimated by a NanoDrop spectrophotometer (Thermo Scientific) and integrity was checked by running it on a 1% Tris-EDTA agarose gel.

Approximately 500 ng of total RNA was used per sample for cDNA synthesis. DNAse treatment and first strand synthesis were performed as described in ref. 15. Quantitative real time PCRs (qPCRs) were performed in a total volume of 10 μl with Kapa SYBR Fast qPCR kit (KAPA Biosystems) on an ABI 7500 fast machine operated with ABI 7500 software (Applied Biosystems). Duplicates were performed for each qPCR reaction. Each experiment was performed at least three times with independently isolated RNA samples. *rp49* was used as the internal control. All primer sequences are listed in Supplementary Table 2. A melt analysis was performed at the end of the reaction to ensure the specificity of the product. The fold change of gene expression in any experimental condition relative to wild-type was calculated as 2^−ΔΔCt^, where ΔΔCt = (Ct (target gene) − Ct (rp49))_Expt._ − (Ct (target gene) − Ct (rp49))_Control._

### Western Blot

Approximately 8–10 pupal CNS of appropriate genotype and age were dissected in phosphate buffer saline and homogenized immediately by vortexing in 100 μl lysis buffer (20 mM HEPES, 100 mM KCl, 0.1% Triton-X, 1 mM PMSF). The homogenate (15 μl) was run on an 8% SDS-PAGE gel and then transferred to a nitrocellulose membrane using standard protocols. After overnight incubation in primary antibody at 4 °C and 1 h in secondary antibody, the signal was probed using chemi-luminiscent detection system (ECL, Thermo Scientific). The following antibodies were used: 1° mouse anti-dSTIM antibodies 8G1 and 3C1^60^ at a 1:20 final dilution, 1° mouse anti β-tubulin (Developmental Studies Hybridoma Bank) at 1:5000 and 2° anti-mouse HRP (Cell Signalling Technology) at 1:10000.

### Library preparation and sequencing

Total RNA was isolated from dissected CNS using TRIzol as described above. The RNA was run on a Bio-analyzer chip (Agilent) to ensure integrity. Approximately 500 ng of total RNA was used per sample to prepare libraries using TruSeq RNA Library Prep Kit v2 (Illumina) following manufacturer’s instructions, but with 1/3^rd^ of the kit recommended volumes^61^. The prepared libraries were run on a DNA1000 chip of Bio-analyzer to check their size. Libraries were then quantified by qPCR and run on an Illumina Hiseq2000 platform. Eight samples were run in a single lane. Biological duplicates were performed for each sample.

### RNA-seq data analysis

Reads obtained after sequencing were aligned to the *Drosophila* genome dm3 release using TopHat^62^. The mapped reads measured to an average coverage depth (C) of 5.8 (±0.7) times the *Drosophila* genome calculated as C = LN/G where L is the length of the reads which in this case was 50, N = number of reads and G is the size of the haploid genome, which for *Drosophila* is 130 Mb.

Differential expression upon *dStim* knockdown was estimated by CuffDiff2, a software package that takes the reads aligned by Tophat^62^ as input, and uses geometric normalization on gene-length normalized read counts, a beta negative binomial model for distribution of reads and t-test for calling differentially expressed genes^63^,^64^. We set a corrected p-value, referred to as the q-value cut-off of 0.05, to identify differentially expressed genes by this method.

The number of reads per gene were also calculated independently using the bioconductor package GenomicRanges^65^. These gene counts were then used for differential analysis with EdgeR, an R based bioconductor software that takes in read counts per sample as input, normalizes them using the Trimmed Mean of M-values (TMM) method and then using a negative binomial model employs an exact test to identify differentially expressed genes^21^,^64^. Here, an FDR-corrected p-value of 0.05 was used as cut-off.

A fold change cut-off was not applied and the minimum significant fold change observed was ±0.5. Only genes with non-zero values in both samples were considered. Genes found to be significantly altered by both methods were considered differentially expressed. The scatter plots, density box plot and dendrogram were generated using CummeRbund^20^. Heat maps were generated using Matrix2png^66^. Comparison of gene lists and generation of Venn Diagrams was carried out using Whitehead BaRC public tools (http://jura.wi.mit.edu/bioc/tools/).

### Climbing assay

Flies of either sex were dropped in a glass cylinder in batches of 10. Flies were collected at the bottom of the cylinder by gently tapping it. The flies were monitored for 12 seconds and the number of flies that crossed an 8 cm mark within these 12 seconds was noted manually. Average number of flies that crossed the 8 cm mark in 12 seconds from a minimum of three independent batches was plotted as a bar graph with standard error of mean.

### Generation of the Ral^WT^ transgenic line

*Ral*^*WT*^ was generated by cloning the coding sequence of *Ral* from wild-type flies. RNA was isolated using TRIzol (Ambion) from adult flies. cDNA was synthesized from 1 μg total RNA using 0.25 μg oligo (dT)_12–18_ (Invitrogen) and 40 uM-MLV reverse transcriptase (Invitrogen) along with 10 mM DTT, 20 units RNaseOUT (Invitrogen) and 1 mM dNTPs (Invitrogen) in a total reaction volume of 25 μl. Primers with NotI and KpnI sites were used for PCR with Phusion high fidelity DNA polymerase (New England BioLabs). Primer sequences are listed in Supplementary Table 2. The PCR product was subsequently digested using NotI and KpnI (New England BioLabs) and cloned into the pUAST attb vector^67^ which was then microinjected in embryos to obtain *Ral*^*WT*^ flies.

### Primary neuronal cultures from pupal brains

The protocol for culturing pupal neurons has been modified from^68^. Pupae were aged between 24–48 h and dissected in dissecting saline (DS) containing 137 mM NaCl, 5.4 mM KCl, 170 μM NaH_2_PO_4_, 220 μM KH_2_PO_4_, 33.3 mM glucose, 43.8 mM sucrose and 9.9 mM HEPES at pH 7.4. Each culture dish contained cells from 4–6 CNS. After dissection, CNS were incubated in DS with 50 units/ml of papain activated by cysteine (1.32 mM) for 15–20 minutes at room temperature. Following enzymatic treatment, dissociated CNS were briefly spun down and washed with DDM2 (DMEM/F-12 with GlutaMAX (Gibco) supplemented with 100 units/ml penicillin-streptomycin (Gibco), 10 μg/ml Amphotericin B (Gibco), 20 mM HEPES, 50 μg/ml insulin and 20 ng/ml progesterone). After washing, the CNS were re-suspended in 50 μl DDM2 and triturated gently with a 100 μl pipette tip until disintegrated to tiny lumps. The total volume was made up to 200 μl with DDM2 and plated on a dish made as described in^68^ coated with 0.1 mg/ml poly D-lysine. The cultures were incubated at 25 °C with 5% CO_2_ for 18–20 h before imaging.

### Live Ca^2+^ Imaging

Pupal cultures washed three times with haemolymph like saline (HL_3_) without calcium (70 mM NaCl, 5 mM KCl, 20 mM MgCl_2_, 10 mM NaHCO_3_, 5 mM Trehalose, 115 mM sucrose, 5 mM HEPES, pH 7.2) before imaging to minimize the amount of calcium in the extracellular fluid. The genetically encoded calcium sensor GCaMP6m was used for observing calcium signals. Thapsigargin (Invitrogen) and calcium to a final concentration of 10 μM and 2 mM were added manually at the indicated time points. Images were taken as a time series on an XY plane at an interval of 4 seconds using a 40× oil objective with an NA of 1.3 on an Olympus FV1000 inverted confocal microscope (Olympus Corp. Japan). The raw images were extracted using Fiji^69^ and regions of interest (ROI) selected using the Time series analyser plugin. ∆F/F was calculated using the formula ∆F/F = (F_t_ − F_0_)/F_0_, where F_t_ is the fluorescence at time t and F_0_ is baseline fluorescence corresponding to the average fluorescence over the first ten frames.

### Statistical Analysis

For all analysis involving more than two test conditions, One-way Analysis of Variance (ANOVA) was performed, followed by pairwise Tukey’s test. Statistical significance post ANOVA is denoted with red alphabets in all figures. In each comparison, conditions with different alphabets are statistically significant at alpha <0.05 whereas conditions with the same alphabet are statistically indistinguishable. Supplementary file 2 contains the exact p-values for all post hoc comparisons. For comparision between two samples, two-tailed unpaired Student’s t-test was used and the p-value is stated in the respective figure legends. All statistical tests were performed using Origin 8.0 software (Micro Cal). Area under the curve calculations were done using Microsoft Excel (Microsoft). All box plots were plotted using BoxPlotR^59^.

### Data

The RNAseq data associated with this manuscript has been submitted to GEO with accession number GSE89168.

## Additional Information

**How to cite this article**: Richhariya, S. *et al*. A pupal transcriptomic screen identifies Ral as a target of store-operated calcium entry in *Drosophila* neurons. *Sci. Rep.* **7**, 42586; doi: 10.1038/srep42586 (2017).

**Publisher's note:** Springer Nature remains neutral with regard to jurisdictional claims in published maps and institutional affiliations.

## Supplementary Material

Supplementary Information

Supplementary File 1

Supplementary File 2
