# Supplementary material for: A pupal transcriptomic screen identifies Ral as a target of store-operated calcium entry in Drosophila neurons
Source: Sci Rep. 2017 Feb 14;7:42586. doi: 10.1038/srep42586 (PMC5307359; doi:10.1038/srep42586)
Supplement: Supplementary Information [file srep42586-s1.doc]

**Supplementary Information**

**A pupal transcriptomic screen identifies Ral as a target of store-operated calcium entry in *Drosophila* neurons**

Shlesha Richhariya, Siddharth Jayakumar, Katharine Abruzzi, Michael Rosbash and Gaiti Hasan

**
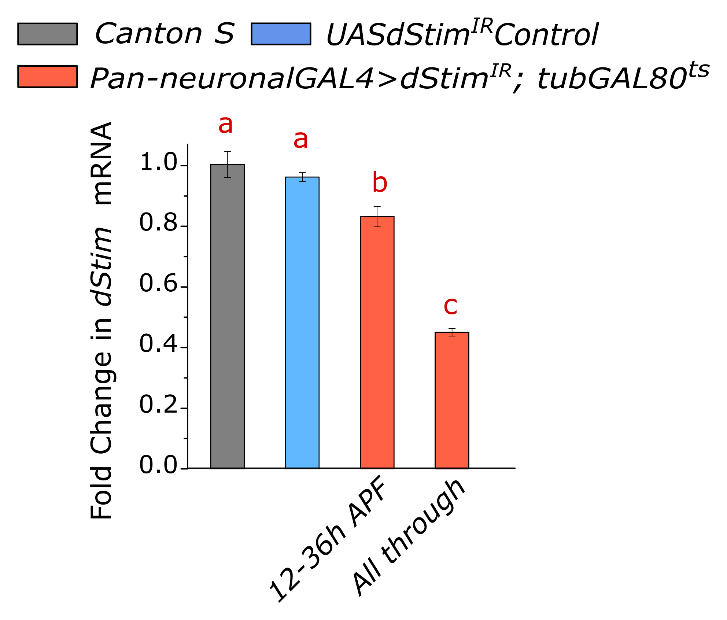
**

**Figure S1: *dStim* RNA levels post knockdown**

Bars indicate mean fold change in *dStim* RNA levels (± SEM) isolated from central nervous systems (CNS) of 36h old pupae of the indicated genotypes. *dStim* knockdown was for the indicated durations. Red alphabets over the bar graphs represent statistically indistinguishable groups (one-way ANOVA with a post hoc Tukey’s test p<0.05).


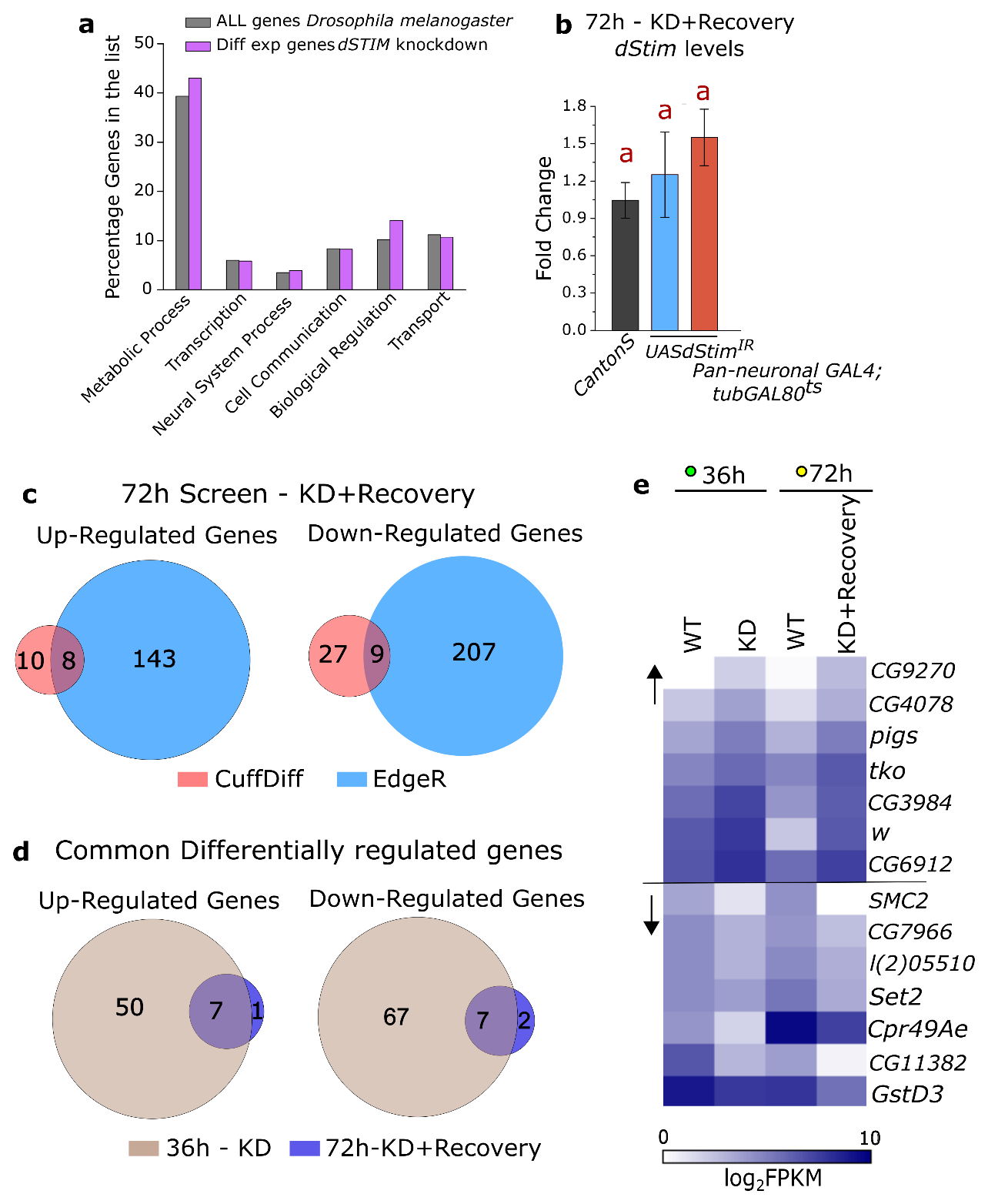


**Figure S2: Altered gene expression upon *dStim* knockdown and recovery**

(a) Bars represent the percentage of all differentially expressed genes at 36h APF upon *dStim* knockdown from 12-36h APF and all genes in *Drosophila* in the indicated biological process by Gene Ontology classification. (b) Bars represent mean (± SEM) of fold changes in *dStim* RNA from the CNS of 72h old pupae of the indicated genotypes measured by qPCR. *dStim^IR^* pupae were subjected to 24h of *dStim* knockdown followed by 36h of recovery. Red alphabets over the bar graphs represent statistically indistinguishable groups (one-way ANOVA with a post hoc Tukey’s test p<0.05). (c) Venn Diagrams representing the number of up and down regulated genes at 72h APF, respectively as quantified by CuffDiff and EdgeR. (d) Venn Diagrams representing the number of common up and down regulated genes between pupae at 36h (*dStim* knockdown) and 72h (*dStim* knockdown + recovery). (e) Heatmap with normalized read counts of 14 differentially regulated genes, identified by both Cuffdiff and EdgeR, at the two time points of 36h (KD) and 72h (KD + recovery).


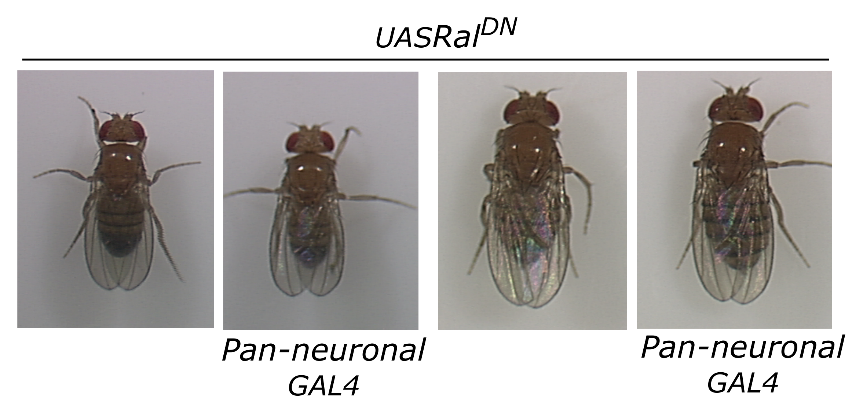


**Figure S3: Neuronal Ral function is not important for gross wing morphology** Images are from male and female flies of the indicated genotypes.


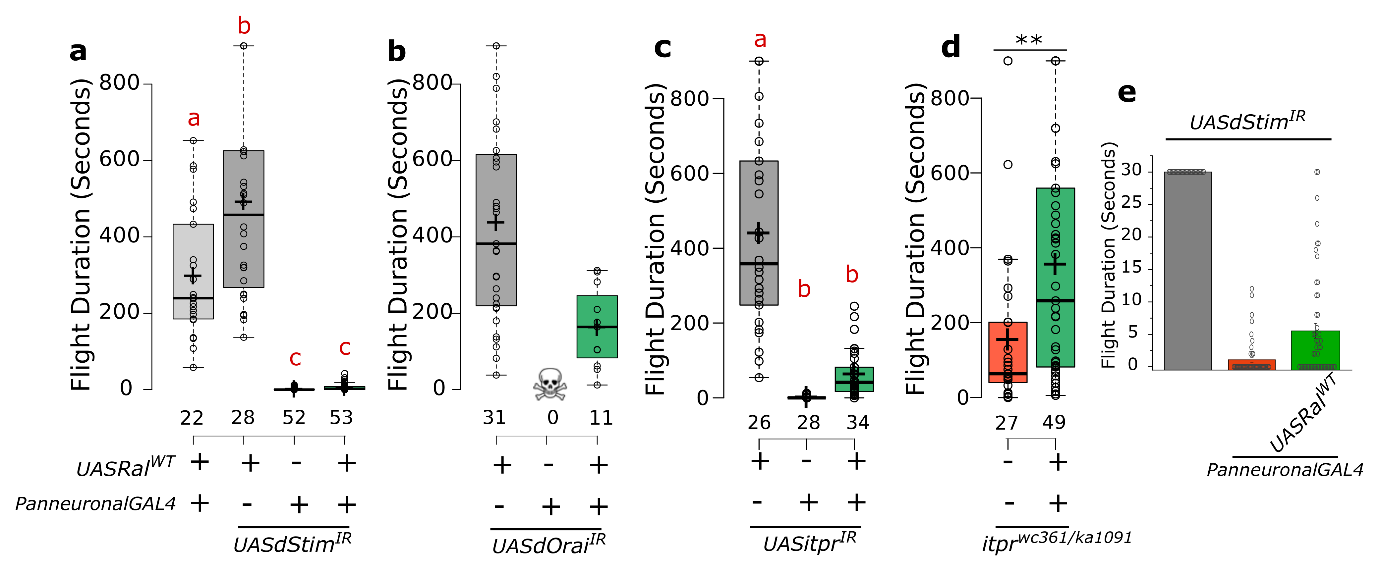


**Figure S4: Ral acts downstream of intracellular calcium signalling to regulate flight**

(a-d) Duration of flight bouts of male flies from the indicated genotypes represented as box plots. (c) Bars represent the flight duration of *dStim* knockdown and *Ral* rescue flies from (a) for the first 30 seconds as mean (± SEM). Open circles are individual data points. Red alphabets over the box plots/ bar graphs represent statistically indistinguishable groups (one-way ANOVA with a post hoc Tukey’s test p<0.05). **p=0.00125, two-tailed t-test.

Supplementary Table 1: List of Fly Stocks

| **Fly line** | **Description** | **Source** |
| --- | --- | --- |
| *elav^C155^ GAL4* | Pan-neuronal Driver | BDSC BL458 ^1^ |
| *Ral^EE1^* | Ral mutant | BDSC 25095 |
| *UASRal^DN^* | UAS-dominant negative of Ral | BDSC 32094 |
| *UASRal^IR^* | UAS-RNAi against *Ral* | BDSC 29580 |
| *GCaMP6m* | UAS-genetically encoded calcium sensor | BDSC 42748 |
| *UASdStim^IR^* | UAS-RNAi against *dStim* | VDRC v47073 |
| *UASdOrai^IR^* | UAS-RNAi against *dOrai* | VDRC v12221 |
| *UASitpr^IR^* | UAS-RNAi against *itpr* | NIG 1063-R2 |
| *tubulinGAL80^ts^* | Expresses GAL80 under the tubulin promoter, two insertions on the II chromosome | Albert Chiang  (NCBS, TIFR, India) |
| *itpr^ka1091^* | *itpr* mutant | BDSC 30739 ^2^ |
| *itpr^wc361^* | *itpr* mutant | 2 |
| *Orai^3^* | *dOrai* mutant | BDSC 17538 |

Supplementary Table 2: List of Primer Sequences

| **name** | **Sequence 5’>3’** |
| --- | --- |
| *Rala_cloning_F* | TTATAAGCGGCCGCATGAGCAAGAAGCCGACAGC |
| *Rala_cloning_R* | TGCGATGGTACCCTAAAGTAGGGTACACTTAAGTCTC |
| *rp49_F* | CGGATCGATATGCTAAGCTGT |
| *rp49_R* | GCGCTTGTTCGATCCGTA |
| *dStim_F* | GAAGCAATGGATGTGGTTCTG |
| *dStim_R* | CCGAGTTCGATGAACTGAGAG |
| *Ral_F* | GACTACGAGCCCACCAAG |
| *Ral_R* | CGGCATAATCCTCCTGGC |
| *dOrai_F* | GAGATAGCCATCCTGTGCTGG |
| *dOrai_R* | CGGATGCCCGAGACTGTC |
| *CG1732_F* | GAGGAGGAGATGTGTGTAAGC |
| *CG1732_R* | TGTCCGTAGTTGTAGGTGAGC |
| *set2_F* | CCAATGCCACCGAGTGTTAC |
| *set2_R* | TCCTTGCGATACGGACGC |
| *CG13897_F* | GACGCCCTTCCAGAATGTC |
| *CG13897_R* | GCTGCTGTCGTTGCTCAG |
| *tko_F* | TGCCAGCTCTAGGACAGC |
| *tko_R* | TTGTAGTACCAGCCGTCGC |
| *path_F* | TACTACAGAACTCGCCGCAC |
| *path_R* | CAGACCAAACAGGATGGAGAAC |
| *gt_F* | AAGACTCCCTCGCCGTATC |
| *gt_R* | GTGGCTTGCTCCTGCTTG |

**Supplementary File legends:**

**Supplementary File 1.** List of genes with altered expression levels upon *dStim* knockdown

**Supplementary File 2.** File with p-values for all ANOVA and post-hoc comparisons

**Supplementary References:**

1. Lin, D. M. & Goodman, C. S. Ectopic and increased expression of Fasciclin II alters motoneuron growth cone guidance. *Neuron* **13,** 507–523 (1994).

2. Joshi, R., Venkatesh, K., Srinivas, R., Nair, S. & Hasan, G. Genetic Dissection of itpr Gene Function Reveals a Vital Requirement in Aminergic Cells of Drosophila Larvae. *Genetics* **166,** 225–236 (2004).
